# Supplementary material for: How do social networks, perception of social isolation, and loneliness affect depressive symptoms among Japanese adults?
Source: PLoS One. 2024 Apr 24;19(4):e0300401. doi: 10.1371/journal.pone.0300401 (PMC11042698; doi:10.1371/journal.pone.0300401)
Supplement: Attachment — Submitted filename: Response to Reviewers.docx [file pone.0300401.s003.docx]

**Response to academic editor and reviewers**

**Journal Requirements:**

**When submitting your revision, we need you to address these additional requirements.**

**1. Please ensure that your manuscript meets PLOS ONE's style requirements, including those for file naming. The PLOS ONE style templates can be found at**

**https://journals.plos.org/plosone/s/file?id=wjVg/PLOSOne_formatting_sample_main_body.pdf and**

**https://journals.plos.org/plosone/s/file?id=ba62/PLOSOne_formatting_sample_title_authors_affiliations.pdf**

Response:

We have now revised the PLOS ONE’s style requirement, to ensure that the new version of the manuscripts fulfils these requirements.

**2. Thank you for stating the following financial disclosure:**

**“This work was supported by JST RISTEX “SOLVE for SDGs: Preventing Social Isolation & Loneliness and Creating Diversified Social Networks” Grant Number JPMJRX21K2, Japan.”**

**Please state what role the funders took in the study. If the funders had no role, please state: "The funders had no role in study design, data collection and analysis, decision to publish, or preparation of the manuscript."**

**If this statement is not correct you must amend it as needed.**

**Please include this amended Role of Funder statement in your cover letter; we will change the online submission form on your behalf.**

Response:

We have described the role of the funders below.

“This work was supported by JST RISTEX “SOLVE for SDGs: Preventing Social Isolation & Loneliness and Creating Diversified Social Networks” Grant Number JPMJRX21K2, Japan. The funders had no role in study design, data collection and analysis, decision to publish, or preparation of the manuscript.”

**3. Thank you for stating the following in the Acknowledgments Section of your manuscript:**

**“This work was supported by JST RISTEX “SOLVE for SDGs: Preventing Social Isolation & 303 Loneliness and Creating Diversified Social Networks” Grant Number JPMJRX21K2, Japan.”**

**We note that you have provided additional information within the Acknowledgements Section that is not currently declared in your Funding Statement. Please note that funding information should not appear in the Acknowledgments section or other areas of your manuscript. We will only publish funding information present in the Funding Statement section of the online submission form.**

**Please remove any funding-related text from the manuscript and let us know how you would like to update your Funding Statement. Currently, your Funding Statement reads as follows:**

**“This work was supported by JST RISTEX “SOLVE for SDGs: Preventing Social Isolation & Loneliness and Creating Diversified Social Networks” Grant Number JPMJRX21K2, Japan.”**

**Please include your amended statements within your cover letter; we will change the online submission form on your behalf.**

Response:

Thank you for pointing this out. We have removed the text regarding funding from the manuscript and revised the Funding Statement below.

“This work was supported by JST RISTEX “SOLVE for SDGs: Preventing Social Isolation & Loneliness and Creating Diversified Social Networks” Grant Number JPMJRX21K2, Japan. The funders had no role in study design, data collection and analysis, decision to publish, or preparation of the manuscript.”

**4. We note that you have stated that you will provide repository information for your data at acceptance. Should your manuscript be accepted for publication, we will hold it until you provide the relevant accession numbers or DOIs necessary to access your data. If you wish to make changes to your Data Availability statement, please describe these changes in your cover letter and we will update your Data Availability statement to reflect the information you provide.**

Response:

Our data use statement remains unchanged. We understand that the manuscript will be held until the accession number or DOI required to access the data is provided.

**5. Please include captions for your Supporting Information files at the end of your manuscript, and update any in-text citations to match accordingly. Please see our Supporting Information guidelines for more information: http://journals.plos.org/plosone/s/supporting-information.**

Response:

We appreciate your pointing this out. The Supporting Information file caption is included at the end of the manuscript.

**See the next page for responses to Reviewer 1.**

**Responses to Reviewer #1:**

**Thank you for providing us with an opportunity to revise this manuscript.**

**Comment: Major Revision.**

**Detailed information:**

Response:

Thank you for providing us with your insights. We appreciate the opportunity to revise the manuscript in detail. We have modified the entire manuscript as you suggested. Based on your comments, we have made the following revisions.

**Abstract
Objective: Need a simple sentence to summarize the background and why this research is being conducted.**

Response:

Thank you for your comment. We have included a sentence summarizing the research background and why we conducted this research, as shown below.

**“**Understanding the interplay between social isolation, loneliness, and the perception of social isolation on depressive symptoms is crucial for effective intervention planning” (pp1, line 3-4).

**Methods：What kind of model is built using structural equations.**

Response:

Thank you for your comment. We have incorporated your comments as shown below.

**“**We assessed the participants’ social network size using the Lubben Social Network Scale, perceived social isolation with a 1-item question, loneliness with the 3-item UCLA Loneliness Scale, and depressive symptoms with the Patient Health Questionnaire-9. Structural equation modeling employed to test the hypothesized model for the effects of social networks, the perception of social isolation, and loneliness on depressive symptoms.” (pp1, lines 9-13)

**Conclusions: How did the new term’ social support’ suddenly appear in the conclusion section, which seems abrupt? Please present important conclusions based on the research purpose and results.**

Response:

Thank you for your comment. We agree with your comment and have changed the term from ‘social support’ to ‘social network,’ as shown below.

**“**It is vital to consider both the quality of social networks and the nature of individual relationships in addition to expanding a person’s social network.” (pp1, line 22-23)

**Introduction
Page 3, Line 80: A very low-level error occurred. The first letter of a word after a colon does not need to be capitalized. If you want to express the former, it’s best not to use ‘first’ to avoid making people think it’s the first difference.**

Response:

We thank you for the careful review. While reviewing the text, we removed this sentence.

**Page 3, Line 83: Why use “Additionally” to connect the two sentences before and after? The expression is incorrect because you are explaining the “effect” in the previous sentence.**

Response:

Thank you for your comment. We agree with you and removed the word ‘Additionally’

**Page 3, Line 89-95: Why is the first sentence in this paragraph contradictory to the following content? Please pay attention to logic and language expression.**

Response:

Thank you for your comment. The passage you pointed out was intended to argue that the correlation between social isolation and loneliness is weak and that social isolation and loneliness do not necessarily co-occur. To clarify our point, we have rewritten the passage paying attention to logic and language expressions as shown below.

**“**Notably, previous research has shown only a weak or modest correlation between social isolation and loneliness [14], indicating that having many objective social connections do not inherently negate feelings of loneliness [15]. Therefore, viewing social isolation and loneliness as conceptually distinct constructs and independently assessing them is critical [6].” (pp2, line 47-51)

**Page 4, Line 111-117: As an outcome variable, the explanation of depression is too brief. Please add relevant introductions.**

Response:

Thank you for your suggestion. We have added an introduction to previous studies to further emphasize that depressive symptoms are a significant outcome of problems such as social isolation and loneliness, as shown below.

**“**Social isolation, acting as a direct stressor, elevates brain stress responses [12], and long-standing depression has been partially explained by social isolation [18]. Prior research indicates that indicators of social isolation leading to depression encompass living alone, possessing a fragile social network, and sparse social interaction [19,20]. Traditionally, loneliness has been intrinsically linked with depression [21], even surpassing objective metrics of social connectedness as a predictive factor for depression [22, 23]. Irrespective of age, profound loneliness associated with severe depressive symptoms [24,25]. Moreover, the perceived lack of societal support and social isolation can lead to depressive symptoms, hindering depressed patients’ recovery [26]. Thus, while many studies have highlighted the relationship between social isolation, loneliness, and perception of social isolation, limited research has concurrently treated these elements as discrete concepts and investigated their serial influence on depression.” (pp3, line 60-70)

**Page 4, Line 125: A brief explanation of the content of Figure 1 needs to be added here.**

Response:

Thank you for your comment. We have briefly explained the model in Figure 1, as shown below.

“Based on antecedent research, we formulated a hypothetical (Fig 1) to illuminate the effect of social isolation (quantified by social network size), its perceptions, and loneliness on depressive symptoms. Table 1 shows a concise summary of these characteristics. Our proposed model postulates that the size of social networks, perceptions of social isolation, and loneliness all converge to influence depressive symptoms. Additionally, perceptions of social isolation and loneliness potentially mediate the connection between social networks and depressive symptoms.” (pp3, line 77-78, pp4, line 79-85)

**Overall: The introduction section on social isolation and loneliness is verbose and lacks logic, please refine it.**

Response:

Thank you for your comment. We reviewed the logical development of the rest of the Introduction and completely revised the Introduction based on your suggestion.

**Methods
Page 6, Participants and Procedures: What is your sample collection method? How do you consider the size of the sample size, and how do you handle samples with unknown gender or ‘other’?**

Response:

Thank you for your comment. We have included specific description of the sample collection method.

“This study was conducted in collaboration with the online research company, Cross Marketing Inc., spanning the period of March 23 to 28, 2022. Cross Marketing Inc. Participants were recruited through a website from the 3.55 million monitor base of Cross Marketing Inc., targeting Japanese individuals aged 20 and above. This recruitment procedure ensured targeting a broad spectrum of respondents throughout Japan. The survey was completed when we received responses from 3,500 participants.” (pp5, line 95-99)

The sample size of this study is larger than in other studies using structural equation modeling. We obtained as large a sample as possible to examine trends across Japan. However, e large samples make significant results more likely. Therefore, caution should be exercised in interpreting the results. We have mentioned this in the manuscript, as shown below.

“We specifically sought a larger sample size to identify trends in pertinent to the Japanese population.” (pp5, line 100)

“Given the large sample size, there is an increased likelihood of obtaining statistically significant results when employing structural equation modeling, hence requiring careful interpretation of findings.” (pp6, line 1844, pp7, line 145-146)

Moreover, as mentioned earlier, this study aimed to examine trends in Japan as a whole. Therefore, we conducted the analysis without excluding participants with unknown gender or other gender participants. We described the treatment of unknown gender and others, as indicated below.

“In the initial phase, for a comprehensive assessment of the hypothetical model, the analysis incorporated data from participants with gender identified as unknown or other.” (pp7, line 147-148)

**Page 6, Measures, Line 155-157: It should be the data result indicating that the internal consistency is good. Please express the semantics more rigorously. Also, what does A refer to? The full name should be written out. Finally, reference literature is needed to prove the source and credibility of this value.
Page 6, Measures, Line 164: Same problem as above.
Page 6, Measures, Line 168: Same problem as above.**

Response:

We appreciate these helpful suggestions. A specific explanation of Cronbach’s alpha, an indicator of the scale's reliability, was missing in the original version of the manuscript. Therefore, we have added a description of Cronbach’s alpha and the references, as shown below.

“We evaluated the reliability of each scale by determining Cronbach's alpha, a widely recognized measure of reliability [36]. An alpha value of 0.70 or higher is deemed acceptable [37].” (pp6, line 132-134)

Thank you for your comment. As shown below, we also included Cronbach’s alpha ratings for each scale in the results to facilitate the reader’s understanding.

“Cronbach's alphas for of the scales were adequate because they were greater than .70.” (pp7, line 158-159)

**Results
Page 4, Line 206-208: So what variables are the results of the values in Table 2? Please describe clearly and write it down in Table 2.**

Response:

Thank you for your comment. We appreciate this suggestion. Table 2 has been renumbered from Table 2 to Table 3 to reflect other additions. Table 3 shows the values of the path coefficients obtained for the hypothetical model by gender from structural equation modeling. We have described the meaning of Table 3 and how to read the table, as shown below.

“The coefficients in Table 3 represent the paths derived from the structural equation modeling. As an illustration, the values in Table 3 suggest that both male and female participants exhibited a significant negative path from family social isolation to perceived social isolation.” (pp9, line 193-196)

**Page 4, Line 209-212: This paragraph should be explained in the statistical analysis section of the methods section.**

Response:

I agree with your opinion. We have removed the paragraph from the relevant section and included the text in the Statistical Analysis section, as shown below.

“We conducted mediation analysis using the Bootstrap method to quantify indirect effects within the SEM model. Calculating the bias-corrected Bootstrap confidence intervals involved 5,000 Bootstrap resamples (with a 95% confidence interval).” (pp7, line 151-153)

**Overall: For the convenience of readers, explanations for variable names in the table can be added in the comments below, such as “LSNS-6 Family” and “R-UCLA.” It is necessary to explain what they represent again.**

Response:

Thank you for your suggestions. We have described each variable under Tables 2 and 3, as shown below.

“LSNS-6 Family; Social isolation from the family, LSNS-6 Friend; Social isolation from friends, Perception of Social Isolation; An individual’s perceptions of their social isolation degree, R-UCLA; Level of loneliness, PHQ-9; Level of depressive symptoms” (pp8, line 172-174, pp10, line 216-218)

**In general, 1) Please highlight important information. 2) Strengthen logic. The reader’s thinking should be guided by you, rather than having the reader struggle to clarify your logic on their own. 3) The English expression of the entire text is problematic and needs to be repaired. 4) The differences between various independent variables need to be clearer to avoid confusion.**

Response:

Thank you for taking the time to review our manuscript. We have revised the overall structure of the discussion and highlighted important information. In addition, the structure of the Introduction and Discussion was modified to strengthen the logic. We reviewed and revised the English expressions throughout the text. We have also included Table 1, summarizing the terms at the end of the introduction to clarify the differences among the independent variables. Additionally, for consistency, we changed the title depression to depressive symptoms. In addition, the variable names in Fig. 1 have been changed to avoid confusion for the reader.

Table 1. Characteristics of social isolation, perceptions of social isolation, and loneliness.

| Variable | Definition and Characteristics |
| --- | --- |
| Social isolation | An *objective* state of having few social contacts with others.  A typical indicator of social isolation is the network size. |
| Perception of social isolation | An individual's perception of the extent of his or her social isolation.  It is an individual's cognitive evaluation, which is different from the number of networks. |
| Loneliness | The lack of social connections (social loneliness) or negative feelings (emotional loneliness) that arise when the quantity or quality of relationships with a particular partner or peer is *subjectively* deficient compared to the person's ideal |

(pp4, line 87-88)

TITLE: “How can we reduce the effects of social isolation and loneliness on depressive symptoms? Findings from an online survey of a Japanese population”

**Fig 1. Hypothetical model of this study.** The squares indicate a psychological variable, and the arrows indicate associations among variables.

**See the next page for responses to Reviewer 2.**

**Responses to Reviewer #2:**

**It is my understanding that this study is the result of an analysis of how social networks and the perception of social isolation and loneliness affect depression among participants of an Internet survey in Japan.

Clarifying the relationship of social isolation and loneliness in the structure of depression would be an important factor to consider in depression prevention measures.

However, this study has serious problems that cannot be scientifically ignored in order to be published in an international journal.

The biggest problem is that this study uses a sample sampled from an unspecified population using the Internet. While Internet-based surveys have the advantage of being less costly and labor intensive, they have the serious problem that the representativeness of the sample is unknown. There is likely to be a strong bias to participate in survey monitoring, and there may be considerable psychological bias. For conducting research on depression and other mental illnesses, Internet-based surveys are difficult to estimate bias, and it would be difficult to seek generality in their results.

Therefore, this study is considered unsuitable for publication in an international journal because the possibility of scientific inaccuracy cannot be denied.

Publication in a domestic journal would enhance its value.**

Response:

Thank you for providing these insights. Your point is correct, but many studies published in PLOS ONE use structural equation modeling of online surveys (Fischer et al., 2014; Soon et al., 2020; Aljaberi et al., 2022). In addition, our study aimed to understand the associations and mechanisms of different variables and not to determine the prevalence or risk ratio in a given population, which we have clearly stated, as shown below. We considered the title to be misleading and have revised it.

“Consequently, this study aims to clarify the interrelations and underlying mechanisms connecting social isolation, perceptions of social isolation, loneliness, and depressive symptoms.” (pp3, line 75-76)

Also, as you point out, based on STROBE regarding cross-sectional studies, we recognize the potential biases and imprecise representativeness inherent in data of cross-sectional designs, which limits the validity of cross-sectional studies. Therefore, we reviewed the reporting guidelines for cross-sectional designs and described sampling and data representativeness as limitations of this study, as described below.

“Lastly, our online data collection method could induce sampling bias, potentially favoring those with ready internet access or those particularly concerned about social isolation and loneliness.” (pp12, line 265-267)

In addition, we have added the maximum, minimum, kurtosis, and skewness of each variable in Table 2, providing additional information about our sample. We hope that these revisions, based on your valuable feedback will improve the quality of this paper.

**Table 2. Descriptive statistics and correlations between the variables.**

|  |  |  | 1 | | 2 | | 3 | | 4 | | | 5 | |
| --- | --- | --- | --- | --- | --- | --- | --- | --- | --- | --- | --- | --- | --- |
| 1 | LSNS-6 Family | | ― | |  |  |  |  |  | |  |  |  |
| 2 | LSNS-6 Friend | | .43 | ^***^ | ― | |  |  |  | |  |  |  |
| 3 | Perception of social isolation | | -.31 | ^***^ | -.32 | ^***^ | ― | |  | |  |  |  |
| 4 | R-UCLA | | -.29 | ^***^ | -.36 | ^***^ | .71 | ^***^ | ― | | |  |  |
| 5 | PHQ-9 | | -.25 | ^***^ | -.23 | ^***^ | .57 | ^***^ | .61 | | ^***^ | ― | |
| *Descriptive statistics* | | |  |  |  |  |  | |  | |  |  |  |
|  | *Mean* | | 5.10 | | 3.28 | | 2.21 | | 5.03 | | | 5.53 | |
|  | *SD* | | 3.33 | | 3.49 | | 0.92 | | 1.94 | | | 6.24 | |
|  | *Minimum* | | 0 | | 0 | | 1 | | | 3 | | 0 | |
|  | *Maximum* | | 15 | | 15 | | 4 | | | 9 | | 27 | |
|  | *Skewness* | | 0.14 | | 0.85 | | 0.31 | | | 0.65 | | 1.40 | |
|  | *Kurtosis* | | -0.79 | | -0.13 | | -0.74 | | | -0.66 | | 1.47 | |
|  | *Cronbach's αlpha* | | .88 | | .89 | | ― | | .85 | | | .92 | |

(pp8, line 171-174)

**References**

Fischer S, Lemmer G, Gollwittzer M, Nater UM (2014) Stress and Resilience in Functional Somatic Syndromes – A Structural Equation Modeling Approach. PLoS ONE 9(11): e111214. https://doi.org/10.1371/journal.pone.0111214

Soon JM, Wahab IRA, Hamdan RH, Jamaludin MH (2020) Structural equation modelling of food safety knowledge, attitude and practices among consumers in Malaysia. PLoS ONE 15(7): e0235870. https://doi.org/10.1371/journal.pone.0235870

Aljaberi MA, Alareqe NA, Alsalahi A, Qasem MA, Noman S, Uzir MUH, et al. (2022) A cross-sectional study on the impact of the COVID-19 pandemic on psychological outcomes: Multiple indicators and multiple causes modeling. PLoS ONE 17(11): e0277368. https://doi.org/10.1371/journal.pone.0277368
