# Supplementary material for: How do social networks, perception of social isolation, and loneliness affect depressive symptoms among Japanese adults?
Source: PLoS One. 2024 Apr 24;19(4):e0300401. doi: 10.1371/journal.pone.0300401 (PMC11042698; doi:10.1371/journal.pone.0300401)
Supplement: Attachment — Submitted filename: Response to Reviewers.docx [file pone.0300401.s004.docx]

**Response to academic editor and reviewers**

**Journal Requirements:**

**When submitting your revision, we need you to address these additional requirements.**

**1. You made some helpful revisions and good progress on this work. There are still, however, some fairly significant issues with lack of details of some methods, and statements that don't always fit with the study. Please pay particular attention to the requests from both reviewers for more details on the methodology. In addition, both reviewers mentioned statements in the manuscript that don't quite match the study content. That relates to criteria 4 here: https://journals.plos.org/plosone/s/criteria-for-publication**

Response:

We reviewed the manuscript in its entirety and attempted to revise the manuscript with reference to Criterion 4 (https://journals.plos.org/plosone/s/criteria-for-publication).

**See the next page for responses to Reviewer 1.**

**Responses to Reviewer #1:**

Thank you for providing critical insights that helped us to improve this paper. We have revised the manuscript and included the analytical data indicated by you. We made the following revisions based on your comments.

**Abstract
Methods：How do you determine that the surveyed sample is a "representative sample"? Is it sufficiently representative? Can it represent the population of Japan?**

Response:

Thank you for this comment. We have reported the analysis and results of verifying the sample's representativeness in the Result chapter, as follows.

**“**To assess the representativeness of our sample relative to the Japanese population, we conducted chi-square tests for gender and age, using demographic data from the Ministry of Internal Affairs and Communications as of March 2022 as a reference. Notably, our study's sample had fewer participants than expected in their 20s and 70s and more in their 40s and 50s compared to the broader Japanese population. This discrepancy suggests that our data may not entirely reflect the demographic composition of Japan. We acknowledge this as a limitation of our study and will discuss its implications in the discussion. Despite these concerns about representativeness, the collected data were utilized for further analysis because our primary objective was to explore the relationships and underlying mechanisms of the variables.” (pp12, line 209-214).

The result of our analysis indicated that our data are not representative of the Japanese population. Nevertheless, we proceeded with the subsequent analyses because this study was designed to understand the associations and mechanisms of the variables and not to determine prevalence or risk ratios in a specific population. However, we have discussed the issue of sample representativeness as a limitation of this study of online surveys.

“Lastly, the online nature of data collection might lead to sampling bias. The data might not fully represent the Japanese population, notably missing those without internet access or experiencing severe social isolation or loneliness. To enhance these findings, further research should consider conducting in-depth interviews with people facing social isolation or intense loneliness (e.g., individuals who have withdrawn from society) and demographic studies in specific communities” (pp19, line 334-339).

**Conclusions: What are the intervention measures? Please write the key conclusion based on your question: "How can we reduce the effects of social isolation and loneliness on compressive symptoms".**

Response:

Thank you for this comments. Based on your comment, I have included the following critical conclusions in the revised manuscript.

**“**Interventions to effectively decrease social isolation and loneliness and thereby mitigate their impact on depressive symptoms should include cognitive behavioral therapy techniques. These techniques aim to alter negative perceptions of social isolation and loneliness and bolster interpersonal skills. We anticipate future longitudinal studies to further refine and strengthen the model proposed in this study by clarifying causal relations among these variables.” (pp3, line 26-30).

**Methods
1.Participants and Procedures: You still haven't solved the core problem. 1) In the research design stage, the size of the sample size should be calculated. Secondly, how is the study population determined? Why choose this research population? 3) Didn't even collect demographic information?**

Response:

Thank you for your comments. In the revised manuscript, we have included information on determining the sample size and study population. We also created Table 1 to show the participants’ demographic information.

**“**We decided to collaborate with Cross Marketing Inc. because it is the most prominent research company in Japan. The sample size for our study was determined using G*Power 3.1.9.7 [40, 41]. Since the purpose of this study was to examine the hypothetical model in an exploratory manner, the sample size required was calculated to be 779 cases, assuming a two-tailed test for the mother correlation coefficient, an assumed correlation coefficient of 0.10, a significance level of 5%, and a power of 80%. We also planned to conduct structural equation modeling (SEM). However, there is no consensus on pre-calculating the power and sample size for SEM [42]. It is recognized that a small sample size may compromise the accuracy and reproducibility of results of SEM [43]. Therefore, we aimed for the largest feasible within our funding constraints, exceeding 779 participants, to ensure a robust analysis and to capture trends representative of the Japanese population.” (pp8, line 118-131).

Table 1. Respondents’ characteristics. (pp13)

| **Variable** | **N (%)** | **Variable** | **N (%)** |
| --- | --- | --- | --- |
| Gender | | Occupation | |
| Male | 1,806 (54.5%) | Employed | 2,395 (72.2%) |
| Female | 1,494 (45.1%) | Homemaker | 392 (11.8%) |
| Other gender | 7 (0.2%) | Unemployed | 473 (14.3%) |
| Unknown | 8 (0.2%) | Student | 25 (0.8%) |
| Age group | | Other | 30 (0.9%) |
| 20-29 | 155 (4.7%) | Annual house hold income | |
| 30-39 | 409 (12.4%) | < 2.0 million | 291 (8.7%) |
| 40-49 | 890 (26.8%) | < 2.0-3.9 million | 533 (16.1%) |
| 50-59 | 1237 (37.4%) | < 4.0-5.9 million | 566 (17.1%) |
| 60-69 | 549 (16.5%) | < 6.0-7.9 million | 423 (12.8%) |
| 70- | 73 (2.1%) | ≥ 8.0 million | 694 (20.9%) |
| Unknown | 2 (0.1%) | Unknown | 808 (24.4%) |

**2.Measures, Lines 112-117: You have not made any modifications yet. You should add data that can demonstrate the reliability and validity of the scale, and this data should have references.**

Response:

Thank you for your suggestions. I have added the data showing the reliability and validity of the LSNS-6. We assessed the reliability using Cronbach's alpha and validity using confirmatory factor analysis as a two-factor structure, similar to previous studies (Kurimoto et al., 2011). We hope the following information is helpful.

“We first conducted a chi-square test on sociodemographic variables to ensure the sample's representativeness. Next, we conducted a confirmatory factor analysis (CFA) to verify structural validity of the LSNS-6. Based on previous research [47], we analyzed a model where the LSNS-6 Family and LSNS-6 Friend factors were each represented by three items. The model's goodness of fit was assessed using several metrics: the chi-square (χ2) test statistic, the Root Mean Squared Error of Approximation (RMSEA), the Comparative Fit Index (CFI), and the Tucker-Lewis Index (TLI). A non-significant χ2 test statistic implies an acceptable fit, but it is essential to note its sensitivity in large sample sizes [48]. CFI and TLI values above 0.95 indicate a good model fit [49], whereas RMSEA values below 0.05 suggest an optimal fit, values between 0.05 and 0.08 a moderate fit, and values between 0.08 and 0.10 a marginal fit [50]. The reliability of each scale was determined using Cronbach's alpha, a widely recognized measure of reliability [51]. An alpha value of 0.70 or higher is considered acceptable [52].” (pp10, line 171-182)

**“**We conducted a confirmatory factor analysis to test the structural validity of the LSNS-6. The model demonstrated a good fit, evidenced by χ2 (8) = 81.03, p < .001, with RMSEA = .05, CFI = .99, and TLI = .99. This confirmed the validity of the two-factor structure for our study's data. We calculated the Cronbach's alpha coefficient to be 0.88 for LSNS-6 Family and 0.89 for LSNS-6 Friend, indicating appropriate reliability for both scales.” (pp13, line 218-222).

**See the next page for responses to Reviewer 3.**

**Responses to Reviewer #3:**

Thank you for providing critical insights that helped us to improve this paper. We have revised the manuscript and included the analytical data indicated by you. We made the following revisions based on your comments.

**I appreciate the opportunity to review the manuscript titled "How can we reduce the effects of social isolation and loneliness on depressive symptoms: Findings from an online survey of a Japanese population." This study investigates the correlation between social isolation, loneliness, and perceived social isolation in connection with depressive symptoms in the adult Japanese population. The authors conducted an online survey among 3315 representative Japanese adults to investigate these connections. The survey incorporated the Lubben Social Network Scale to gauge the size of the participants' social networks, the UCLA Loneliness Scale consisting of 3 items to evaluate loneliness, and the Patient Health Questionnaire-9 to measure depressive symptoms. In particular, the extensive sample size of 3315 participants enhances the robustness of the findings. The authors employed structural equation modelling to examine their hypothetical model, which considered the impact of social networks, perception of social isolation, and loneliness on depressive symptoms. The results revealed that larger social networks exhibited a weak correlation with lower levels of loneliness and depressive symptoms. Specifically, the study indicated that perceived social isolation was associated with increased levels of both loneliness and depressive symptoms.
In general, this study has considerable merit. To further enrich the scientific value of the manuscript, I have outlined some key points for the authors' consideration based on the feedback from the two reviewers. My aim is to ensure a comprehensive understanding of the comments of the reviewers, to evaluate the adequacy of the authors' responses, and to provide additional suggestions to enhance the manuscript.**

Response:

Thank you very much for your evaluation of this paper. We appreciate the opportunity to revise the manuscript in detail. We have modified the entire manuscript as you suggested. Based on your comments, we have made the following revisions.

**TITLE
1. While the current title of the study provides some information on its focus, I would recommend a more descriptive one such as "Examining the Impact of Social Networks and Perceived Social Isolation on Depressive Symptoms among Adults in Japan". At its current stage, it would be premature to conclude that the study aims to mitigate the negative impact of social isolation and loneliness on depressive symptoms.**

Response:

Thank you for your suggestion. As you suggested, we changed the title to be more descriptive.

**“**How Social Networks, Social Isolation and Loneliness Effect on Depressive Symptoms among Japanese Adults?” (pp1, line 4-6).

**ABSTRACT
The abstract provides a comprehensive overview of the study. However, a few improvements could enhance its clarity and impact.

2. Please consider changing (“We assessed the size of the social network size using the Lubben Social Network Scale”) to (“We used the Lubben Social Network Scale to determine the size of the social network”).**

Response:

Thank you for your comments. We have revised the abstract according to your suggestion.

“We used the six-item Lubben Social Network Scale to assess the size of their social networks.” (pp2, line 9-10)

**3. The statistical significance of the goodness of fit of the model should be emphasized. Providing a brief interpretation of the beta coefficients and their implications for the interaction between social isolation, loneliness, and depressive symptoms would make the results more accessible to a wider audience.**

Response:

Thank you for your valuable advice. In the revised manuscript, I described that the model had a good fit, as shown below.

“The final model demonstrated a satisfactory fit with the data (χ2 (1) = 3.73; not significant; RMSEA = 0.03; CFI = 1.00; TLI = 1.00).” (pp2, line 16-17).

We also determined that it is appropriate to include the interpretation of the beta, or path coefficient, in the statistical analysis section described in the text below. We also stated that the chi-square value is an indicator of model fit sensitive to the sample size.

“A non-significant χ2 test statistic implies an acceptable fit, but it is essential to note its sensitivity in large sample sizes [48]. CFI and TLI values above 0.95 indicate a good model fit [49], whereas RMSEA values below 0.05 suggest an optimal fit, values between 0.05 and 0.08 a moderate fit, and values between 0.08 and 0.10 a marginal fit [50].” (pp10, line 176-180)

**4. The conclusion could be strengthened by reiterating the practical implications of the study findings and how they can contribute to the development of effective intervention strategies. Furthermore, providing some suggestions for future research directions can add depth to the conclusion.**

Response:

Thank you for your comments. We have revised our conclusions as follows.

“Interventions to effectively decrease social isolation and loneliness and thereby mitigate their impact on depressive symptoms should include cognitive behavioral therapy techniques. These techniques aim to alter negative perceptions of social isolation and loneliness and bolster interpersonal skills. We anticipate future longitudinal studies to further refine and strengthen the model proposed in this study by clarifying causal relations among these variables.” (pp3, line 26-30)

In the discussion, we included details on the practical implications of this study and its contribution to developing effective strategies.

“However, studies on the effectiveness of such preventive measures are limited, underscoring the need to develop intervention programs focusing on primary prevention against social isolation and loneliness. Our study suggests developing early psychoeducational programs for school education and community-based cognitive-behavioral therapeutic interventions to prevent social isolation and loneliness. Such initiatives could play a vital role in preventing depressive symptoms arising from social isolation and loneliness.” (pp19, line 322-327)

**INTRODUCTION**

**5. Although the study's hypothetical model is briefly mentioned, providing a more detailed integration of the model within the context of the existing literature could strengthen the background and set the stage for the study's aims and objectives.**

Response:

Thank you for your very useful suggestions. I have added a more detailed explanation of the hypothetical model and included the reasons for using perceived social isolation and loneliness as mediating factors.

“From existing literature, we hypothesize that having more of social networks is negatively related to perception of social isolation, loneliness, and depressive symptoms [24, 27, 28]. We expected that perceptions of social isolation, which arise from individuals evaluating their social networks, positively related to loneliness and depressive symptoms [24, 25, 34]. Consistent with its established link to depression [29], we expected loneliness to relate positively to depressive symptoms. Further, we suggest that perceptions of social isolation and loneliness might mediate the relationship between social networks and depressive symptoms, as these perceptions often have a significant impact on mental health than objective social isolation measure [25, 30, 31].” (pp6, line 97-104)

**6. I suggest narrating the terms in the text and providing relevant literature instead of intruding on a table to define variables, as it breaks the conventional approach.**

Response:

Thank you for your suggestion. We have removed Table 1 and modified it to include subheadings to explain the terms in the text with related references.

**7. The relevance of this entry is not clear (Fig 1. Hypothetical model of this study. The squares indicate a psychological variable and the 1 arrows indicate associations among variables.)**

Response:

Thank you for pointing this out. We have revised Figure 1 to reflect the positive and negative predictions of the path coefficients between each variable.

**Fig 1. Hypothetical model of this study.** In this diagram, squares represent different psychological variables. Arrows between these squares depict the relationships among these variables. Dashed arrows illustrate negative, and solid arrows indicate positive paths.

**8. The authors did mention some of the expressions of loneliness, including hikikomori. There is an additional concept that correlates with loneliness that needs to be mentioned: social avoidant personality disorder, Taijin kyofusho, Modern Type Depression, Unemployment, and Not in Employment Education or Training (NEET). The economic and demographic factors that contribute to increased loneliness in Japan need to be further elaborated.**

Response:

Thank you for your thought-provoking comment. I have included additional information on the concepts and economic and demographic factors contributing to the increase in social isolation and loneliness in Japan.

“Factors such as social anxiety, avoidant personality disorder, and modern depression increasingly linked to heightened social isolation and loneliness in Japan [3-5], where these issues are more pronounced than in other cultures [6, 7]. Specifically, social anxiety disorder and avoidant personality disorder are believed to lead to societal withdrawal, especially among young people [8]. Additionally, the rise of individuals Not in Employment Education or Training (NEET) and unemployment -related economic challenges are associated with increasing social isolation and loneliness in Japan [9, 10].” (pp4, line 38-44)

**METHOD**
**9. In the abstract, we were told 'Lubben Social Network Scale… with a 1-item question' and in the method, we were told (“The LSNS-6 comprises six items…”.). Please reconcile.**

Response:

Thank you for pointing this out. I made the following revisions to maintain consistency.

“We used the six-item Lubben Social Network Scale to assess the size of their social networks. We employed a single question to gauge perceived social isolation. Loneliness was assessed using the three-item UCLA Loneliness Scale, and depressive symptoms were assessed using the Patient Health Questionnaire-9.” (pp2, line 9-12)

**10. The authors have described the outcome measures under the subheading "Measures". However, it may be necessary to mention the psychometric properties of the Japanese version of these measures. On the other hand, in line 131, pg. 6, it was stated that “Initially, we computed descriptive statistics for LSNS-6 Family, LSNS-6 Friend, Perception of Social Isolation, R-UCLA, and PHQ-9. We evaluated the reliability of each scale by determining Cronbach's alpha, a widely recognized measure of reliability [36]. An alpha value of 0.70 or higher is deemed acceptable [37]”. Exercises to explore reliability should be detailed in the subheading ‘Measures’ and removed from the subheading ‘statistical analysis’. Some of the literature cited appears to allude that the Japanese reliability versions of these instruments. Indeed, the authors did cite the relevant study but did not narrate the issue of the Japanese version of The LSNS-6 (Kurimoto A, Awata S, Ohkubo T, Tsubota-Utsugi M, Asayama K, Takahashi K, Suenaga K, Satoh H, Imai Y. [Reliability and validity of the Japanese version of the abbreviated Lubben Social Network Scale]. Nihon Ronen Igakkai Zasshi. 2011;48(2):149-57. Japanese. doi: 10.3143/geriatrics.48.149.**

Response:

Thank you for your comments, we have added the confirmatory factor analysis data to the Results section to demonstrate the reliability and validity of the LSNS-6. We have also included Cronbach's alpha coefficients for each scale in the Results section, as we believe it is appropriate to include Cronbach's alpha coefficients for assessing reliability along with the results of the confirmatory factor analysis.

“We first conducted a chi-square test on sociodemographic variables to ensure the sample's representativeness. Next, we conducted a confirmatory factor analysis (CFA) to verify structural validity of the LSNS-6. Based on previous research [47], we analyzed a model where the LSNS-6 Family and LSNS-6 Friend factors were each represented by three items. The model's goodness of fit was assessed using several metrics: the chi-square (χ2) test statistic, the Root Mean Squared Error of Approximation (RMSEA), the Comparative Fit Index (CFI), and the Tucker-Lewis Index (TLI). A non-significant χ2 test statistic implies an acceptable fit, but it is essential to note its sensitivity in large sample sizes [48]. CFI and TLI values above 0.95 indicate a good model fit [49], whereas RMSEA values below 0.05 suggest an optimal fit, values between 0.05 and 0.08 a moderate fit, and values between 0.08 and 0.10 a marginal fit [50]. The reliability of each scale was determined using Cronbach's alpha, a widely recognized measure of reliability [51]. An alpha value of 0.70 or higher is considered acceptable [52].” (pp10, line 171-182)

“We conducted a confirmatory factor analysis to test the structural validity of the LSNS-6. The model demonstrated a good fit, evidenced by χ2 (8) = 81.03, p < .001, with RMSEA = .05, CFI = .99, and TLI = .99. This confirmed the validity of the two-factor structure for our study's data. We calculated the Cronbach's alpha coefficient to be 0.88 for LSNS-6 Family and 0.89 for LSNS-6 Friend, indicating appropriate reliability for both scales. Also, the Cronbach's alpha coefficients for R-UCLA and PHQ-9 were 0.85 and 0.92, respectively, both of which were sufficiently reliable.” (pp13, line 218-223)

**11. Although the method states that efforts were made to ensure diversity in the participant pool, it would be beneficial to provide more explicit details on the specific measures taken to ensure representation across various sociodemographic factors, such as age, location (rural and urban), socioeconomic status, and cultural background. Including a breakdown of these demographics in the participant pool can add credibility to the study's results.**

Response:

Thank you for your critical comments. We have presented the demographic data in Table 1.

Table 1. Respondents’ characteristics. (pp13)

| **Variable** | **N (%)** | **Variable** | **N (%)** |
| --- | --- | --- | --- |
| Gender | | Occupation | |
| Male | 1,806 (54.5%) | Employed | 2,395 (72.2%) |
| Female | 1,494 (45.1%) | Homemaker | 392 (11.8%) |
| Other gender | 7 (0.2%) | Unemployed | 473 (14.3%) |
| Unknown | 8 (0.2%) | Student | 25 (0.8%) |
| Age group | | Other | 30 (0.9%) |
| 20-29 | 155 (4.7%) | Annual house hold income | |
| 30-39 | 409 (12.4%) | < 2.0 million | 291 (8.7%) |
| 40-49 | 890 (26.8%) | < 2.0-3.9 million | 533 (16.1%) |
| 50-59 | 1237 (37.4%) | < 4.0-5.9 million | 566 (17.1%) |
| 60-69 | 549 (16.5%) | < 6.0-7.9 million | 423 (12.8%) |
| 70- | 73 (2.1%) | ≥ 8.0 million | 694 (20.9%) |
| Unknown | 2 (0.1%) | Unknown | 808 (24.4%) |

**12. Although the method briefly mentions that participants were informed about the study objectives and ethical considerations, it would be advantageous to provide more detailed information on the informed consent process. Specifically, explaining how participants were informed about their rights, the purpose of the study, the possible risks and benefits, and how their data would be used and protected can help ensure transparency and build trust with participants.**

Response:

Thank you for your suggestion. We have added details of the informed consent procedure as follows.

“Before commencing the survey, they were informed that their responses would remain anonymous and that there would be no negative consequences for choosing not to respond or withdrawing from the survey at any point. It was explained that all survey responses would be processed statistically, ensuring no individual identification. Furthermore, strict protocols were followed in handling all responses, from the processing stage to data storage and eventual disposal. Participation was entirely voluntary and based on the informed consent of the participants.” (pp8, line 133-138)

**13. Although the method mentions that participants were given reward points as a token of appreciation, it might be useful to elaborate on the nature of these incentives and how they were determined. Providing information on how the incentive value was determined, whether it was proportional to the time or effort required, and how the reward system was designed to prevent bias or undue influence can improve the transparency of the recruitment process.**

Response:

Thank you for your comment. I have described the reward points given to participants.

“They received reward points from the survey agency as a gesture of gratitude for their time and input. These points, determined by the number of questions answered, are a unique feature of Cross Marketing and could be exchanged by participants for goods or cash.” (pp8-9, line 139-141)

**14. Although the method briefly mentions the exclusion of entries with missing or incomplete information, it would be beneficial to include a more detailed description of the data quality control measures implemented throughout the study. Describing the specific criteria used to exclude incomplete or unreliable data, as well as the steps taken to verify the precision and consistency of the collected data, can help ensure the reliability and validity of the study findings.**

Response:

Thank you for the valuable suggestion. I have described the criteria for excluding incomplete data as follows.

“The criteria for exclusion included responses that were evidently dishonest (for example, identical numerical answers for all survey items) and missing responses to the scales measuring social networks, perceptions of social isolation, loneliness, and depressive symptoms.” (pp9, line 143-145)

**The statistical analysis approach described appears to be comprehensive and well-structured. Some points to consider:
15. While you mentioned that you refined the model by eliminating non-significant paths, it would be beneficial to provide a more detailed explanation of the criteria you used for determining the significance of these paths. Clearly defining the thresholds or criteria used for the inclusion or exclusion of paths would enhance the transparency of the model refinement process.**

Response:

Thank you for the useful comment. We described the exclusion criteria for non-significant paths as shown below. We also described the excluded paths from the hypothetical model through structural equation modeling.

“The threshold for statistical significance of path coefficients was set at 5%. Our analysis revealed that the path linking social isolation from friends to depressive symptoms was not statistically significant, leading to its exclusion from the model.” (pp14, line 242-245)

**16. In your description of the mediation analysis using the Bootstrap method, it would be helpful to provide more context on the specific mediators considered and the rationale behind their selection. Furthermore, explaining how the Bootstrap method was used to quantify indirect effects and the specific variables tested for mediation can add depth to the analysis process.**

Response:

Thank you for this comment. I added a detailed explanation of the bootstrap method to the statistical analysis section.

“We also performed a mediation analysis using the Bootstrap method to assess the indirect effects within the SEM framework. This method takes a sample of researchers of size N and creates a new sample from which to extract the replacement N values of the independent, mediating, and dependent variables. For example, this option can be repeated 5000 times to compute 5000 indirect effect estimates [54]. The Bootstrap method is known for providing accurate confidence intervals for indirect effects, as these are based on the empirical distribution of the estimates [55, 56].” (pp11, line 198-202)

In addition, I provided an explanation of the rationale for considering social isolation and loneliness as mediating factors in the Introduction.

“Further, we suggest that perceptions of social isolation and loneliness might mediate the relationship between social networks and depressive symptoms, as these perceptions often have a significant impact on mental health than objective social isolation measure [25, 30, 31].” (pp6, line 101-104)

**DISCUSSION**

**In the discussion section of your manuscript, you effectively summarised the findings and their implications. However, there are a few areas where the discussion could be improved:**

**17. While briefly touching on the potential implications of the findings, it would be beneficial to provide a more detailed discussion on how these results can be applied practically in the field. Describe how understanding the interaction between social networks, perceived social isolation, loneliness, and depressive symptoms can inform the development of targeted interventions and support programs for people experiencing mental health challenges.**

Response:

Thank you for your comment. We have reiterated the mechanisms identified in this study and included a discussion of the potential application of these results.

“Our findings revealed that the direct effect of social network size on loneliness and depressive symptoms was marginal. However, comprehensively analyzing social networks, perceptions of social isolation, and loneliness and examining their effects on depressive symptoms identified a mechanism in which the relationship between social network size and depressive symptoms was mediated by the perceptions of social isolation and loneliness that increase feelings of loneliness and depressive symptoms. This result extends the findings of previous studies that examined these variables in isolation [24-26, 29-34].” (pp18, line 288-294)

“While most studies on social isolation and loneliness have focused on older adults [37], the findings of this study are significant for showing that social isolation and loneliness can cause mental health decline, regardless of age or gender, underscoring the universal applicability of our findings.” (pp18, line 299-302)

**18. Highlight the novel contributions of your research in the context of the existing literature. Clearly articulate how your study contributes to the current understanding of the relationship between social networks, perceived social isolation, loneliness, and depressive symptoms, especially in the context of implications for mental health interventions. Discuss how your findings provide a nuanced understanding that can inform the development of more effective and targeted strategies to address mental health challenges related to social isolation and loneliness.**

Response:

Thank you for your valuable comment. We have revised our discussion to describe the novelty of this study clearly. We have also described the implications of our findings for developing strategies and programs for social isolation and loneliness.

However, comprehensively analyzing social networks, perceptions of social isolation, and loneliness and examining their effects on depressive symptoms identified a mechanism in which the relationship between social network size and depressive symptoms was mediated by the perceptions of social isolation and loneliness that increase feelings of loneliness and depressive symptoms. This result extends the findings of previous studies that examined these variables in isolation [24-26, 29-34].” (pp18, line 289-294)

“While most studies and intervention methods on social isolation and loneliness have focused on older adults [37, 57, 58], the findings of this study are significant for showing that social isolation and loneliness can cause mental health decline, regardless of age or gender, underscoring the universal applicability of our findings.” (pp18, line 299-302)

**19. Based on the findings of your study, provide clear and actionable recommendations for researchers, policymakers, and mental health professionals. Discuss specific strategies and interventions that can be implemented to mitigate the adverse effects of perceived social isolation and loneliness on depressive symptoms. Include suggestions for future research directions and potential areas for further exploration in this field.**

Response:

Thank you for your comment. Based on the research findings, we have described specific interventions and strategies to reduce or prevent the impact of social isolation and loneliness on depressive symptoms.

“Our findings underscore the importance of addressing the transition from perceptions of social isolation and loneliness to depression. Interventions aimed at altering perceptions of social isolation can help reduce depressive symptoms and alleviate loneliness if they target cognitive and emotional aspects across various age groups. Psychoeducational approaches and cognitive-behavioral therapy for changing cognitions and emotions alleviate loneliness effectively [36]. These interventions can be pivotal in transforming perceptions and reducing loneliness and depressive symptoms. Additionally, reminiscence-based psychological interventions have shown effectiveness [57]. We advocate for strategies focusing on the cognitive and emotional facets of social isolation and loneliness rather than merely expanding social networks [39] because social isolation does not necessarily lead to loneliness [23].

Previous research underscores the importance of primary prevention in addressing social isolation and loneliness [60-62]. However, studies on the effectiveness of such preventive measures are limited, underscoring the need to develop intervention programs focusing on primary prevention against social isolation and loneliness. Our study suggests developing early psychoeducational programs for school education and community-based cognitive-behavioral therapeutic interventions to prevent social isolation and loneliness. Such initiatives could play a vital role in preventing depressive symptoms arising from social isolation and loneliness.” (pp18-19, line 311-327)

**20. Although you acknowledge the limitations of the study, it would be valuable to provide a more comprehensive discussion of the implications of these limitations for the interpretation and generalisation of the results. Discuss the potential impact of cross-sectional design, self-reported data, and online data collection method on the validity and reliability of the findings. In addition, provide suggestions for future research that can address these limitations and provide more robust evidence**.

Response:

Thank you for pointing out the limitations of this study. We have discussed the limitations of interpreting and generalizing the results. We have also added suggestions for future studies to provide more robust evidence.

“Lastly, the online nature of data collection might lead to sampling bias. The data might not fully represent the Japanese population, notably missing those without internet access or experiencing severe social isolation or loneliness. To enhance these findings, further research should consider conducting in-depth interviews with people facing social isolation or intense loneliness (e.g., individuals who have withdrawn from society) and demographic studies in specific communities.” (pp19, line 334-339)

**REFERENCE**

**21. The authors included 39 references. Most of them are recent and relevant.**

Thank you for your observation. We have included more references as we revised the manuscript.

**LANGUAGE AND GRAMMAR**

**22. There are issues with expressions and syntax. Ensure that the language used is clear and easily understandable to a wider audience. Try to simplify complex sentences and use straightforward language where possible.**

Thank you for taking the time to review our manuscript. We have revised the English expressions and syntax in the text to make it as straightforward as possible so that many readers can easily understand it.

We thank you again for allowing us to improve our manuscript with your valuable comments and queries. We have worked hard to incorporate your feedback and hope these revisions will persuade you to accept our manuscript for publication in PLOS ONE.
