# Supplementary material for: How do social networks, perception of social isolation, and loneliness affect depressive symptoms among Japanese adults?
Source: PLoS One. 2024 Apr 24;19(4):e0300401. doi: 10.1371/journal.pone.0300401 (PMC11042698; doi:10.1371/journal.pone.0300401)
Supplement: Attachment — Submitted filename: Response to Reviewers.docx [file pone.0300401.s005.docx]

**Response to academic editor and reviewers**

**Journal Requirements:**

**Please review your reference list to ensure that it is complete and correct. If you have cited papers that have been retracted, please include the rationale for doing so in the manuscript text, or remove these references and replace them with relevant current references. Any changes to the reference list should be mentioned in the rebuttal letter that accompanies your revised manuscript. If you need to cite a retracted article, indicate the article’s retracted status in the References list and also include a citation and full reference for the retraction notice.**

Response:

We have verified that the reference list is complete. We have changed the following reference to a more suitable one.

Before change

21. Lin N., Ensel W. M., Simeone R. S., & Kuo W. Social support, stressful life events, and illness: A model and an empirical test. Journal of Health and Social Behavior. 1979; 20(2): 108-19. Doi: 10.2307/2136433.

After change

21. Cacioppo JT, Hawkley LC, Ernst JM, Burleson M, Berntson GG, Nouriani B, et al. Loneliness within a nomological net: An evolutionary perspective. Journal of Research in Personality. 2006; 40(6): 1054-1085. doi:10.1016/j.jrp.2005.11.007

**Additional Editor Comments:**

**Thank you for addressing feedback well on this revised manuscript. There is some need for language revision to improve flow and readability. Some editing by a native speaker appears all that is needed. That will only require an editor and not a full review.**

Response:

Thank you for your appreciation of our response. The manuscript was proofread by a native speaker to improve flow and readability. We have also attached a proofreading certificate for your review.

**See the next page for responses to Reviewer 1.**

**Responses to Reviewer #1:**

Thank you for reviewing our revised manuscript. We have made the following revisions to the manuscript with regard to the areas you have indicated.

**Abstract**

**Conclusions: Please use shorter language to explain, this is an abstract and not a summary.**

Response:

Thank you for pointing this out. We have revised the description to be shorter.

“Results indicate that interventions of psychological approaches, such as cognitive–behavioral therapy, are effective in reducing the perception of social isolation and loneliness, which may lead to the prevention of depressive symptoms. Future longitudinal studies are expected to refine and strengthen the proposed model.” (pp2, line 21-24).

**There are no other objections, however, this paper needs to be thoroughly edited by native English-speakers.**

Response:

Thank you for your confirmation. The manuscript has been entirely proofread by a native speaker and the problem areas have been corrected. We have also attached a proofreading certificate for your review.

**See the next page for responses to Reviewer 3.**

**Responses to Reviewer #3:**

**Thank you for reconsidering to re-review the manuscript titled "How Social Networks, Social Isolation, and Loneliness Affect Depressive Symptoms Among Japanese Adults." I appreciate that the authors have incorporated all of my suggestions, and overall the manuscript is commendable. It seems ready for acceptance with only a few minor revisions. Great job!**

Response:

Thank you very much for evaluating our work. We deeply appreciate your suggestions to further improve our manuscript. We have revised the manuscript as follows with regard to the points you have highlighted.

**TITLE**

**The title is generally clear and conveys the main topics of the study. However, there are a couple of grammatical issues that could be addressed for clarity. Use of "effect" vs. "affect": The correct term to use in this context is "affect" rather than "effect." "Affect" is a verb that means to produce a change or influence, while "effect" is a noun referring to the change or result in itself. Therefore, the title should be: Revised Title: "How Social Networks, Social Isolation, and Loneliness Affect Depressive Symptoms among Japanese Adults."**

Response:

Thank you for highlighting this grammatical problem. We have revised the title as per your suggestion.

“How Do Social Networks, Perception of Social Isolation, and Loneliness Affect Depressive Symptoms among Japanese Adults?”

**ABSTRACT**

**For consistency, you may want to ensure that you use similar terms throughout the title. For instance, you used "loneliness," but in the abstract, the term "perceived social isolation" is mentioned. Depending on the specific focus of your study, you might want to use consistent terminology.**

Response:

Thank you for your valuable suggestions. In our research, we treat “perceived social isolation” and “loneliness” differently. Therefore, we have changed the term “social isolation” in the title to “Perception of social isolation”.

“How Do Social Networks, Perception of Social Isolation, and Loneliness Affect Depressive Symptoms among Japanese Adults?”

In addition, the terms “perceptions of social isolation” and “perceived social isolation” in the abstract were revised to “perception of social isolation” to make them consistent.

“This study aims to elucidate the complex relationship among social isolation, loneliness, and perception of social isolation and its influence on depressive symptoms by evaluating a hypothetical model.”（pp2, line3-5）

“We employed a single question to gauge perception of social isolation.”（pp2, line8-9）

**INTRODUCTION**

**Briefly elaborate on the "8050 problem" to provide readers with a clearer understanding of this specific societal challenge in Japan.**

Response:

Thank you for your suggestion. We have added an explanation of the 8050 issue.

“A particularly acute issue, which is known as the 8050 problem, involves octogenarian parents caring for their socially reclusive children aged in their 50s [1]. In this problem, socially withdrawn children become middle-aged, and the parents who have cared for them typically become elderly, such that caring for their children becomes increasingly difficult. This issue is not unique to Japan; instead, it is a grave one internationally [2] and measures are required to address this issue.” （pp3, line29-34）

**Otherwise, it should be ready to be accepted and may have a more thorough check of grammar and syntax before publication.**

Response:

Thank you for your careful review of our manuscript. It was thoroughly proofread by a native English speaker for grammar and syntax. We hope you will also review the proofreading certificate.

We thank you again for allowing us to improve our manuscript with your valuable comments and queries. We have worked hard to incorporate your feedback and hope that these revisions will persuade you to accept our manuscript for publication in PLOS ONE.
